# The overall structure of the multi-domain amyloid precursor protein

**DOI:** 10.1186/1750-1326-8-S1-P55

**Published:** 2013-10-04

**Authors:** Ina Coburger, Sven O Dahms, Manuel E Than

**Affiliations:** 1 Leibniz Institute for Age Research – Fritz Lipmann Institute (FLI), Jena, Germany

## Background

The amyloid precursor protein (APP) is a type I transmembrane protein that is expressed in a wide number of different cell types. Proteolytic processing by beta- and gamma-secretases releases 38-43 amino acid long peptides, so called Aβ amyloid peptides that accumulate within the plaques in the brain of Alzheimer´s disease patients. Alternatively, initiation of the proteolysis cascade by alpha-secretase prevents the development of these toxic peptides [1,2].

In spite of intense research regarding the involvement of APP in Alzheimer´s disease, The three-dimensional structure of the entire protein, its physiologic function and the regulation of its proteolytic processing remain, however, largely unclear to date [3].

## Materials and methods

To gain a deeper understanding about it, we cloned and recombinantly expressed different constructs of APP in *E. coli*. Using limited proteolysis followed by mass spectrometry and Edman degradation as well as analytical gel permeation chromatography coupled static light scattering, we experimentally analyzed the structural domain boundaries. Using, pull-down assays and analytical gel filtration experiments we analyzed whether different domains interact with each other.

## Results

We experimentally determined that the large ectodomain of APP consists exactly of two rigidly folded domains – the E1- and the E2-domain. The acidic domain, connecting E1 and E2, as well as the juxtamembrane region, connecting E2 to the single transmembrane helix, are highly flexible and extended. In addition, we demonstrated that the E1-domain does not tightly interact with the E2-domain, both in the presence and in the absence of heparin.

## Conclusions

APP hence forms an extended molecule that is flexibly tethered to the membrane. Its multi-domain architecture enables together with the many known functionalities the concomitant performance of several, independent functions, which might be regulated by cellular, compartment specific pH-changes.
